# Understanding Sociocultural Influences on Physical Activity in Relation to Overweight and Obesity in a Rural Indigenous Community of Fiji Islands

**DOI:** 10.1007/s40615-022-01336-0

**Published:** 2022-06-08

**Authors:** Kamal Nand Singh, Marguerite C. Sendall, Philp Crane

**Affiliations:** 1 School of Health, Federation University, 333 Ann St, Brisbane, QLD 4000 Australia; 2 Department of Public Health, College of Health Sciences, Qatar University, Doha, Qatar; 3 Faculty of Arts, Business and Law, School of Social Sciences, University of Sunshine Coast, Maroochydore DC, Locked Bag 4, Brisbane, QLD 4558 Australia

**Keywords:** Health promotion, Physical activity, Overweight and obesity, Culture, Indigenous, Community-based participatory research, Sociocultural

## Abstract

**Introduction:**

Given health disparities and increased rates of obesity and non-communicable diseases seen in Indigenous populations worldwide and the evidence connecting sociocultural knowledge with physical activity, health, and wellbeing, this research was undertaken to understand the social and cultural components contributing to obesity in the Indigenous Fijian rural areas.

**Methods:**

This research is a community-based participatory research (CBPR) project, which engaged community members from a rural iTaukei village in the Fiji Islands. Data collection was carried out through community consultation and semi-structured interviews. The data was analysed using descriptive thematic analysis.

**Results:**

Four major themes emerged associated with sociocultural, economic, political, and physical environmental factors. Males emphasised sports and working on farmlands as preferred types of physical activity, while females focused on family activities and daily activities and support for females’ separate playgrounds. There was a focus on previous health promotion programs that did not incorporate the cultural values, cultural competence beliefs, and traditional ways of the rural Indigenous Fijian community.

**Conclusion:**

The healthcare providers and policymakers need to recognise the iTaukei community culture and appreciate traditional methods to promote equitable community participation in decision-making for health promotion. These findings should inform future research and community-based health programs to address the physical activity levels of the rural Indigenous community and may be relevant to other Indigenous peoples.

## Introduction

The Republic of Fiji, in line with other developing Pacific Island nations, has seen a significant rise in the prevalence of non-communicable diseases (NCDs), which have become the primary cause of death [1]. Proportional mortality in the Fiji Islands increased between 1960 and 2000 from around 20% to more than 45% of all cardiovascular and other NCD deaths in adults [1]. Physical inactivity is one of six contributing risk factors that cause the most disease burden in the Indigenous Fijians (hereafter respectfully referred to as iTaukie) [2]. At the same time, NCD development is complex, the importance and potential impact of increased physical activity. Therefore, the iTaukie community, as part of efforts to improve Indigenous health and wellbeing, suggests that this issue of physical activity be given attention [3]. Adults with high body mass index (BMI) are at risk for NCDs, including hypertension, stroke, cardiovascular diseases, diabetes, and multiple cancers [4]. Studies confirm daily exercise is an effective primary and secondary prevention measure to prevent NCDs [5]. However, overweight and obesity rates are rising across populations where low-income and Indigenous people are at the most significant risk [5, 6]. Some studies have documented an inverse relationship between low socioeconomic status and obesity. However, other studies report that this connection is more complex, particularly among racial and ethnic backgrounds [7]. People with lower socioeconomic status (SES) have, on average, poorer health and die younger than those with more favourable SES. However, overweight and obesity rates do not differ by economic cost in the Pacific region [8]. According to regional differences, current statistics by the World Health Organization indicate 33% of all adults in the Pacific Island region were overweight and obese in 2014, an increase from 29.3% in 2010, including most Indigenous communities [9].

Globally, physical inactivity Interconnects with local environmental factors, which contributes to increase the prevalence of overweight and obesity among people [10]. The association between individual and ecological factors includes genetic makeup and reveals variations in body size among individuals regarding overweight and obesity [11]. Reliable evidence from Fiji and research from other developing countries have shown that Fiji environments, particularly in city regions, are associated with increased rates of physical activity [12]. Regular physical activities and healthy eating can reduce obesity and related NCDs [13]. However, most Pacific Islands do not meet the recommended WHO physical activity guidelines [14]. According to the World Health Organisation (WHO) STEPS Fiji NCD report conducted in 2011, 57.5% of the sample population engaged in high physical activity levels. Significantly more men (72.4%) had a higher participation rate in physical activities than women (42.8%) [15].

Physical activity decreases with ageing and the peak decline from 54 to 64 years. There is a particularly steep decline among iTaukie females compared to their counterparts who are non Indigenous [15]. iTaukie females have the lowest physical activity and the highest sedentary behaviour levels, contributing to increased rates of overweight and obesity [16]. Structural macro-, meso-, and micro-social factors such as colonisation, discrimination, and disparity have impacted iTaukie physical activity [17]. Multiple studies have reported interventions to improve physical activity at the community level, such as the provision of community playgrounds. However, many health promotion initiatives are short lived and do not have the sustainability to develop long lasting impacts on communities [18–20].

Developing and implementing physical activity interventions in Indigenous communities pose numerous challenges. Many Indigenous communities are located in rural and remote regions with limited opportunities or access to recreational facilities [21]. Common environmental barriers to physical activity in rural areas include limited access to facilities, sidewalks, poor lighting/lack of streetlights, uneven road surfaces, wildlife, and inclement weather [22, 23]. These physical environmental barriers are in addition to community, individual level, or personal barriers, including lack of playground, cultural factors, family responsibilities, gender, and socioeconomic factors [19]. In the iTaukie population, colonialism has resulted in Indigenous people being subject to land dispossession, acculturation, political marginalisation, socioeconomic oppression, and health inequities with increased morbidity and decreased life expectancy [24]. Although there are universal commonalities for Indigenous people, each Pacific nation has its unique history shaped by historical, social, political, and cultural factors that significantly influence the contemporary context. Socioeconomic factors contribute to inequity in the determinants of health for Indigenous people, who experience higher rates of morbidity and mortality than their non-Indigenous counterparts [25]. In addition to poorer health outcomes, Indigenous people often have poor utilisation or delayed presentation to the appropriate healthcare facility, increasing the burden of disease and mortality for these vulnerable groups [26]. International literature identifies, most commonly, socioeconomic and environmental factors as the significant barriers to healthcare utilisation and the major contributor to poorer health outcomes [13, 18, 27].

A growing number of community-based participatory research (CBPR) studies show that involving Indigenous communities in the design and implementation of the research and involvement of local Indigenous health care professionals [28, 29]. The CBPR approach emphasises an interactive relationship linking characteristics of the community (e.g., gender, self-efficacy), intrapersonal factors (e.g., family and peers), social (e.g., cultural norms, social support/reinforcement), and physical environment (e.g., built environment) factors can influence behaviour, including physical activity [22, 23, 27, 30–33]. Thus, a more in-depth understanding of determinants and the sociocultural context,, and beliefs can reveal factors that affect physical activity for the iTaukie. Sociocultural context relates to communities’ unique shared values, beliefs, and practices, influencing the acceptance and implementation of health promotion intervention and impacting health-related behaviours [34]. In addition, identifying gaps in knowledge and understanding of what physical activity means to the iTaukie community in rural area? This research also highlights the need for such research on these dynamic topics [10]. The current research examines the sociocultural context that influences the iTaukie communities’ attitudes and beliefs about physical activity. The following research question guided this research: what physical activity means to the iTaukie community in the rural area, recognising a target group’s opinions and perspectives, is crucial when designing and implementing a successful health promotion program among the iTaukei community those residing in the rural setting.

## Methods

### Study Design

This research adopted a CBPR approach to engage community members from a rural iTaukei village on Viti Levu, Fiji Islands. CBPR is a collaborative approach that involves all partners or stakeholders equally in the process and recognises the unique strengths each brings [13]. CBPR begins with a topic relevant to the community and aims to combine knowledge with action and achieve social change [35]. CBPR has been successfully implemented with Indigenous populations in developing countries and informed culturally safe health promotion programs and interventions on a global scale [36, 37].

The first stage of this research involved community consultation and negotiating the research purpose and process. A Health Research Team (HRT) was established during the second stage. It consisted of a principal researcher, local village health worker, community health nurse, primary health medical practitioner, dietitian, community diabetic nurse, and sub-divisional community health charge nurse. The third stage involved data collection and analysis, dissemination of findings, and community feedback in confirming the findings.

### Recruitment and Participants

The participants were recruited from a rural iTaukei village. There were three inclusion criteria for participants — [1] rural Indigenous iTaukei, [2] aged 18 years and over, and (3) individuals who have a BMI higher than or equal to 25 kg/m^2^ (i.e., overweight or obese) [38]. Thus, a convenience purposeful sample for this current study was undertaken [39]. These inclusion criteria ensured a broad range of iTaukei cultural perceptions, which could be explored. Of the 26 overweight and obese iTaukei invited to participate in the study, four overweight and ten obese participants agreed to be interviewed. Twelve people declined to be interviewed or were not contactable.

### Data Collection

The HRT, led by the principal researcher, collected the data. The principal researcher was born in Fiji to an iTaukei mother and a Fijian-born, Indian-descent father. Semi-structured interviews were conducted, and the qualitative findings were made available to the iTaukie community. All participants provided written informed consent. Ethics approval was granted by the Queensland University of Technology and adherence to the Fiji National Health Research Guide 2015 [40]. Additional information on survey methodology and sample characteristics can be found elsewhere [13]. A semi-structured interview guide was developed based on a literature review, project objectives, and the principal researcher’s emic perspective. All interviews were conducted in English at the participant’s home, audio recorded with permission and ranged from 20 to 45 min. The interview guide included questions about (1) availability of the local environment, (2) what physical activity means and its relevance for healthy eating (the benefits, barriers and influences) and locally available resources, (3) physical activity information available in the community, (4) culture and body image, and (5) physical activity interventions in the village and the improvements which could be made. Prompts were used to ensure deep and rich information of perceptions and experiences gathered from participants [13]. Participation was voluntary and confidential. Data saturation was achieved after 14 interviews whereby no new concepts or ideas emerged, and the findings were confirmed with two community consultation and feedback process using the CBPR approach.

### Data Analysis

Data analysis was undertaken using descriptive thematic analysis [41]. Data analysis involved the following steps: familiarisation, coding, interpreting, and exploring the underlying sociocultural, physical, economic, and political environment linked to overweight and obesity [10, 42]. The transcripts were manually analysed and noted for recurrent themes. For example, the key phrases were systematically examined to identify explanatory accounts, and preliminary typologies were developed [43].

The researcher undertook a judicious process of reflexivity to ensure rigour. The process of reflexivity involves a forensic examination of the research process by the researcher to ensure all research decisions are transparent. [44]. This is crucial in developing quality in research. Three processes were implemented to ensure soundness, trustworthiness, and rigour in the research design and analysis phases. The first process was the development of a discussion guide to ensure a similar range and style of questioning were conducted with each participant. The second process was co-judge concordance undertaken by two other researchers in the research team. Co-judges read three transcripts and agreed on the coding framework [45]. Thirdly, the tentative findings were presented to the community to receive feedback.

## Results

**Fig. 1:**
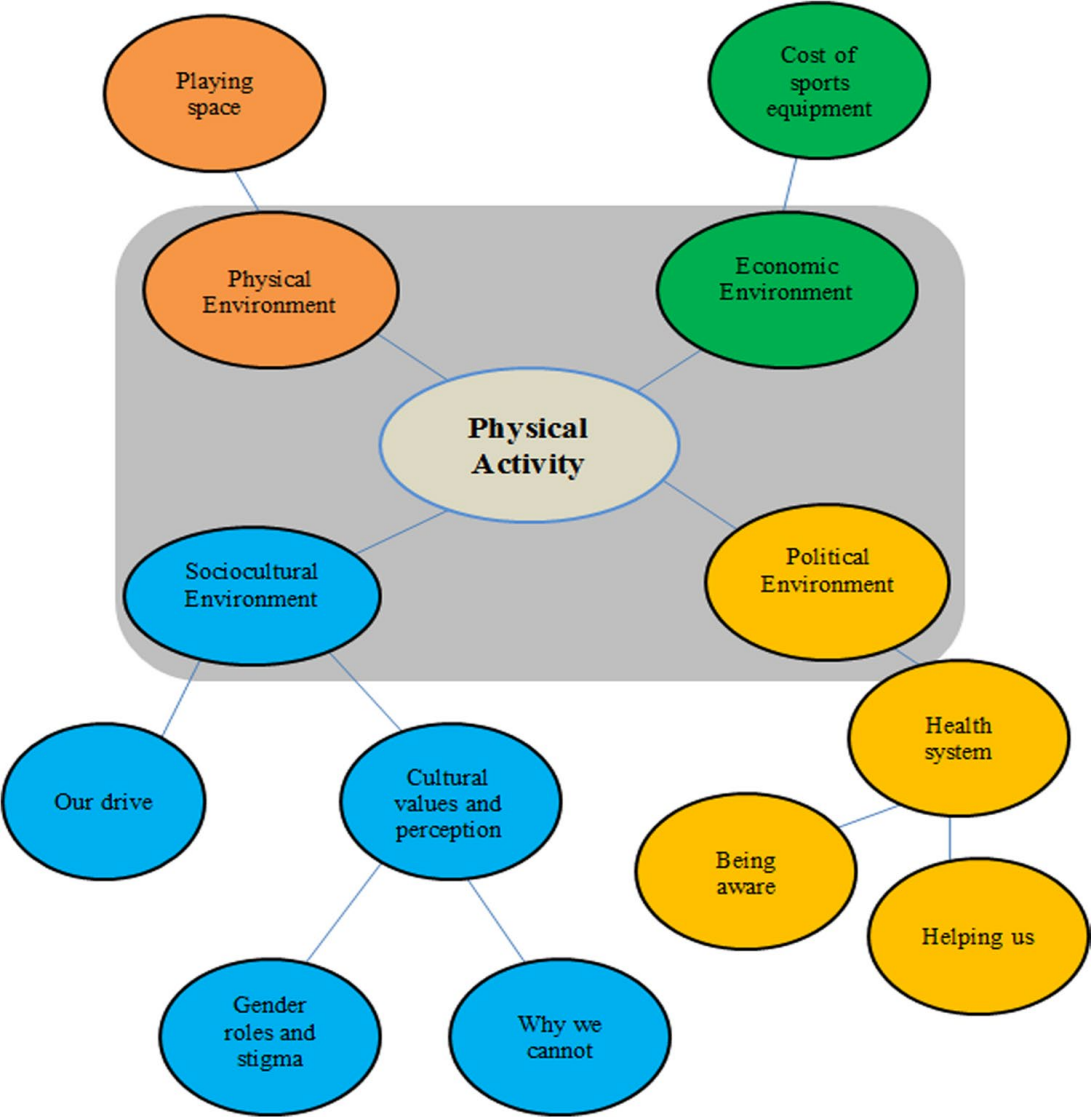
Summary of factors pertaining to physical activity in the rural community environment

The findings from the data analysis using descriptive thematic analysis are categorised under (a) sociocultural, (b) physical, (c) economic, and (d) political environment factors about physical activity in the rural community environment (Fig. 1). Direct quotes from participants provide as evidence of the themes. Participant codes included their initials, age, and overweight (BMI 25–30 kg/m^2^) or obese (BMI over 30 kg/m^2^).

### Sociocultural Environment

#### Our Drive

Unanimous agreement was obtained from all the study participants as they described motivation as one of the most significant barriers. Participants described the need to create awareness among the iTaukei population regarding the benefits of physical activity and the importance of healthy lifestyle changes. One participant explained:

Like we can have a group, a physical activity group, you know a women's group. They can have, maybe … Back in the village, they have their own women's session and if the program of physical activity can also be included in their program especially for them to … or even in the morning, about five o'clock or four o'clock when they can take a morning walk together, everybody as a group. I think that will motivate them rather than be individually it will be more fun too to go as a group. (Participant 3).

The iTaukei community acknowledged that the lack of peer group support influenced individuals’ motivation to adopt healthy eating and increase physical activity. A few participants explained there had been a lack of incentives and a lack of social support from peers to motivate the community when it comes to physical activity, which leads to a lack of self-care.

#### Cultural Values and Perception

##### Gender Roles and Stigma

Participants discussed married females’ feelings of fear of the elders in the village while doing physical activity, resulting in village women staying at home, looking after the family, and cooking for the family rather than participating in any form of physical activity. Participants described these are the traditional roles of women in the village. Here is a comment from one of the participants:

Yes it is a culture for Fijian (iTaukei) you know the girls to stay home, only the boys to go and play. That is our Fijian culture. Once you married, for you to look up to the family the girls. You're not supposed to go to the playground because the playground is only for the men. (Participant 12).

Participants elaborated about women not being allowed to associate with men in the village to play sports. Participants explained the relationship between men and women in the iTaukei kinship culture, where women are not allowed to have direct communication with their uncles (*Momo*). Some participants linked wearing clothes as an issue for the females in the village as they need to wear a long dress (long pants or jeans are not allowed) that will not show their whole body. Therefore, it is difficult for females to play sports or do physical activity with males in the village. Most participants reinforced the above statement and explained how gender segregation is a significant factor. Participants mentioned differences in how physical activity is perceived in the rural villages compared with urban settings, where it as a different way of life.

##### Why We Cannot

The kinship system is an essential aspect of iTaukei life: the ways in which people interact with each other based upon their relationship with each other within the family unit. In the iTaukie community, respect and avoidance relationships are critical to the kinship system. Respect is based on three main concepts: age, gender, and social distance. The older the person is, the more respect they command, regardless of gender or social rank. Male participants explained the existing socially acceptable ways of expressing what it means to be a man or a woman in an iTaukei sociocultural context and how that plays a crucial role in determining access, participation levels, and benefits from being physically active. Female participants’ views were that there is a lack of support for women from the men in the village. The sentiment of the female participants was expressed by one of the participants:

Yes, it is caused by the … our families and sometimes the men of the family they stop the ladies from going to the playground. That is our Fijian culture. Once you married, for you to look up to the family, the girls. You are not supposed to go to the playground because the playground is only for the men. (Participant 12).

Participants perceived families, and the community do not support females doing any physical activity. The female participants confirmed they wanted support from village elders to do physical activity. Due to iTaukei customary practices and respect for elders, they feel isolated, lack freedom, and feel neglected by the community.

### Physical Environment

#### Playing Space

Most participants, especially those with young children, indicated that the lack of facilities and infrastructure prevented children and families from participating in physical activities. Participants mentioned females, in particular, face many challenges in doing physical activity due to a lack of space. Some participants believed the village needs to have its own playground and indoor space for older women’s physical activity. Female participants voiced their concerns that for cultural reasons, they needed a separate playground from the males to retain females’ privacy, which would encourage females of all ages to play sport and be physically active. Currently, some females are reluctant to do physical activity due to environmental and cultural barriers. Participants explained there is a school playground attached to the village with one female participant stating:

Yes, but only in the afternoon after school, which is normally used by men to play rugby, and we women have to look after the kids and cook the dinner for the family. (Participant 1).

Participants elaborated on the lack of space for females to do physical activity by saying he/she felt accessing the playground was unsuitable for females but pointed out that the village could build a playground for females, which the community would greatly value.

### Economic Environment

#### Cost of Sports Equipment

Participants portrayed the opinion that doing physical activity is challenging, with many barriers, including the lack, and high cost of equipment the village cannot afford. The following quote illustrated this:

In the village you know mostly is the financial that is the most problem. So the boys in the community, we have to get money, income to take care of our financial, tuition for the game or … Only the thing happening in the village or we have prepared for the game. Mostly seven aside yeah we have to find our own. Even in the village, little support from the parents but not … only fifty per cent they can support the boys playing rugby and at that time I was being coaching in the top level. I see that most problems in the village are that, less support from the community. I train them, I take them to some of the rugby competition but you see we are the most weak point for rugby what we need resources' we cannot afford it because of lack of support from the community and financial. (Participant 3).

Participants articulated government agencies lack interest in rural areas, and there are no incentives or interventional community programs related to physical activity or sports. Participants stated government agencies had not supported them to maintain and develop facilities in the village. Further, the lack of sports gear is a significant barrier to motivating and encouraging villagers to play sports. Participants conveyed a strong belief national governments have good, structured sports programs. However, they are not being decentralised and or infiltrating to the village level.

### Political Environment

#### Health System

##### Support and Barriers

Participants succinctly verbalised having support from their community and families when it comes to healthy livelihood. For example, one participant made light of the situation by recommending that prominent ex-sports players in the village be good role models and support the village with healthy living. In response, another participant stated village role models are not active because they are not given any incentives from the community or government. One participant suggested:

There should be a good advice given to the youths and people of the village. We elders who are retired sportsperson give them good advice to our young generation to keep physically active so that they can play better sports and go out to play national and international sports so that these young youths can support their family in the village financially. We mostly tell the youths not to consume alcohol, Kava, do not smoke and eat healthy food. We have many rugby players retired who are good role models in our village who come to the school ground during training and give advice to the young youths such as village church minister, village health worker and the chief as well. (Participant 3).

Some of the participants indicated that even though iTaukei health care workers are available at the community health centre, they do not meet community needs and do not understand village cultural needs, as it varies from district to district. During the feedback process, the village pastor commented that the health sector needed to modify the health promotion programs to be culturally appropriate to the specific village. He repeatedly encouraged local health workers to engage with rural communities rather than complete the required paperwork. One participant believed the environment needs to be developed in the community before any health promotion interventional programs are attempted.

##### Being Aware

There was a consensus about the lack of physical education and awareness from local health providers at the community level. The literacy level of the community is low as most villagers have not completed secondary school. Participants’ understanding of physical activity varied, with some stating it meant regular duties, while others defined it as hard labour. Another participant spoke about his/her strong desire for health awareness support from a local health professional in the community and for a place to gather and learn. Also, she/he explained a need for traditional ceremonies to be a part of physical education programs. All of the participants believed there should be a multisector approach to meet the needs of the village, as this woman explained:

We need some adviser for the ministry, for the sports. We need some more workshops to clear things. We don't know most of it, they really hoping the communities know everything but that is not correct. The health department, the youth and sports. They have to come and do some workshop training for the community. (Participant 4).

Participants voiced firm concerns about the lack of support and information on women's health from the community’s health care providers. Participants believed local health providers should come to the community, provide educational resources, and conduct physical activity awareness programs culturally acceptable to the local villagers.

## Discussion

This paper has explored the perception and experiences of the sociocultural context of physical activity linked to overweight and obesity in the rural iTaukie community. It is crucial to capture the views and knowledge of the marginalised community before implementing programs. Economic, political, sociocultural, and physical factors contribute to various health behaviours such as roles in a family, community responsibilities, and an individual’s values, beliefs, and priorities [13, 14, 34, 46]. As identified in this research, iTaukie culture influences physical activity with history and connection to the land, gender roles, and access to facilities in rural environments, contributing to iTaukie healthy activities [22, 27, 47, 48]. Understanding the sociocultural context of physical activity was an essential factor influencing iTaukies’ willingness to participate. Biomedical models of health focus on medically determined negative indicators and outcomes of health, such as ill-health, illness, and disease states, as opposed to broader determinants of health such as client social wellbeing [49]. There is a growing recognition that culturally appropriate health promotion programs that target whole communities are more effective in improving the health status of individuals.

Sociocultural factors are interconnected to iTaukie culture, where females feel singled out from the community, which brings disempowerment and health disparities [50, 51]. Although all genders experience health disparities, health conditions prove one of the most significant barriers to females actively participating in physical activity [52]. Therefore, this matter should not be ignored when targeting this population.

The provision of opportunities for physical activities and sports sketch a discriminated picture for iTaukei, where the rural communities appear to be highly disadvantaged by local government incentives. In contrast, local councils have created recreational parks and footpaths for individuals to engage in physical activity in urban districts. Social isolation is another problem for upland iTaukei people, who are marginalised in many ways because of their traditional culture, customs, and religious beliefs. Rural communities have minimal access to government and financial services, roads, markets, basic education, and health services. Sport is a vital form of physical activity for across ages and gender. It generates success and creates valuable iTaukie role models for families and communities. Many studies reported that being Indigenous, iTaukie people are more likely to have natural athletic ability, which is already a significant aspect of Indigenous men’s lives [4, 8, 13, 27, 53]. ITakuie females in the rural community require specific attention when developing physical activity health promotion programs. With their experience of inequalities and lack of confidence, there is an imperative to develop safe environments for females and develop physical activity programs funded by the government targeting iTaukie women to encourage their participation [54].

Many elderly iTaukie acknowledged they are not as active as they were in their adolescence, and there are not many healthy elderly role models for the upcoming younger generation [8]. Some concerns arise about what encourages and motivates them to maintain physically active in rural iTaukie communities throughout their lifetime. There is a demand for culturally oriented physical activity programs which can positively bring the family and community together [10]. Therefore younger generation will be able to influence and motivate elderly iTaukie in the community. To ensure cultural appropriateness, health promotion physical activity programs targeting rural iTaukie communities should consider these preferences.

Previously, iTaukie had more active lifestyles but due partly to a loss of traditional values and practices such as hunting, lifestyles have become more sedentary. Many Indigenous communities have adopted modern conveniences and ‘western’ lifestyle practices [55, 56]. This research suggests that health policy design primarily involves adopting community consultation methods to utilise the existing population, leading to change. This understanding will help local community health clinicians and the local researchers examine the problem and contribute towards developing long-term solutions.

The iTaukie has identified barriers and enablers that require further exploration: whether health promotion programs demonstrate better outcomes in the short term and are more culturally inclusiveness appropriate in long-term programs. The effectiveness of culturally appropriate physical activity health promotion programs can be measured by increased participation amongst iTaukie children and adults and exploring what interventions are appropriate in engaging physical activity by elderly males and females [57]. We identified rural iTaukei communities had poor participatory rates in sports, serious income inequalities, poor health literacy, gender inequality, a shortage of health-based human resources in various ministerial departments, and a lack of interest in tailoring health programs in a socially acceptable manner. These issues have remained overlooked since the British colonisation era [53]. The authors believe the CBPR approach was culturally appropriate, which also established that people respond positively to success (empowerment, social cohesion, and the power of social reciprocity) [58]. Therefore, understanding the sociocultural factors in the prevalence of obesity and employing a CBPR approach to reinforce achievable changes would highlight a community’s key strengths rather than apportioning blame for its shortcomings.

This research explicitly presented the efficacy of the process of working in consultation with rural villagers to understand the sociocultural factors contributing to physical activity and enable the researchers to study beliefs and practices. In addition, this research addressed the gaps in existing research by detailing the research design, particularly the collaborative process within the iTaukei community, and the development of specific health promotion intervention strategies.

Relatively little is known about the perceptions of iTaukie people regarding physical activity, and there is a dearth of studies from rural communities in particular. Further research across Fiji Islands from these settings is necessary. More research is recommended to understand sociocultural contexts, attitudes, perceptions, and beliefs that iTaukie communities have encompassing physical activity. It is impractical to assume one health promotion program will work the same way for the whole nation. To reduce the gap in health disparities of rural iTaukie communities, this research can inform the development of culturally appropriate health programs which may improve their sustainability. Developing an understanding of sociocultural factors that influence what physical activity means to iTaukei people sheds light on the need for research on these sensitive topics and the design of community health promotion programs.

### Limitation

Conducting CBPR is time-consuming as it involves working with the community to develop an agenda on a regular basis [13]. The obstacles faced in this work were notably similar to those encountered by other researchers [28, 58–60], especially in applying principles of Indigenous research methods and worldviews. A lack of trust can impede researchers from accessing underrepresented communities, deepening community engagement, and forming a true partnership with them [29, 61].

## Conclusion

This research shows sociocultural appropriateness around beliefs, attitudes, and perceptions surrounding physical activity in iTaukie communities. The knowledge and themes drawn from this research have provided answers to understand sociocultural factors contributing toward physical activity in the rural iTaukie communities. The main themes emerging from this research include families and communities, culture and environment, and physical activity with gender differences and several barriers to engaging were also identified.

This research sheds light on differences in perceptions according to gender. iTaukie females have lower participation rates in physical activity; from this research, after gathering their perceptions, healthcare workers and government agencies can work together with the community to alleviate perceived barriers [62]. Female perceptions demonstrate they do not have similar freedoms to partake in physical activity as males. They have family responsibilities, and cultural norms limit their participation [54].

Physical activity was perceived in three ways: structured exercise, incidental exercise, and sports [63]. Physical activity was perceived as a relatively disengaging experience unless done with the same gender and youths, whereas everyday activities were necessary for iTaukie people’s lives. The community perception discussed moving around, being active, and mobile to describe their day-to-day activities. The biomedical model of health promotion programs was not the best approach when developing physical activities in rural iTaukie communities. The Fijian Government and international agencies such as WHO and AusAID, academic institutions, and community health clinicians must support, create, and report on different campaigns to promote physical activity using CBPR principles as an essential mode of delivering health promotion–based programs.

In addition, there is a need to report the shortcomings and setbacks encountered in this approach so that sufficient structural changes can be made as part of community public health programs, which can be adequately resourced. With the help of this research, the CBPR approach has proven to be a helpful step in working with the iTaukei communities. Understanding the sociocultural contexts and perceptions of rural iTaukie communities is essential when developing interventions to increase participation in physical activity, improve wellbeing, and reduce the risk of NCDs if efforts are effective and sustainable. It is highly recommended this is applied to future research with iTaukei communities, particularly with regard to research that focuses on the widening gaps between urban, rural, and remote iTaukei communities.

## Data Availability

The data supporting this study’s findings are openly available in Queensland University of Technology eprints at https://doi.org/10.5204/thesis.eprints.110807, reference number 110807.

## References

[1] Ministry of Health Fiji. National strategic plan 2016–2020 Fiji Islands: Ministry of Health; 2016 [1–31]. Available from: http://www.health.gov.fj/PDFs/Corporate%20Plan/Strategic%20Plan%202016-2020%20Executive%20Version.pdf.

[2] Taylor R, Lin S, Linhart C, Morrell S (2018). Overview of trends in cardiovascular and diabetes risk factors in Fiji. Ann Hum Biol.

[3] Utter J, Faeamani G, Malakellis M, Vanualailai N, Kremer P, Scragg R, et al. Lifestyle and obesity in South Pacific youth: baseline results from the Pacific Obesity Prevention in Communities (OPIC) project in New Zealand, Fiji, Tonga and Australia. 2008.

[4] Gyaneshwar R, Naidu S, Raban MZ, Naidu S, Linhart C, Morrell S (2016). Absolute cardiovascular risk in a Fiji medical zone. BMC Public Health.

[5] Cameron N, Godino J, Nichols JF, Wing D, Hill L, Patrick K (2017). Associations between physical activity and BMI, body fatness, and visceral adiposity in overweight or obese Latino and non-Latino adults. Int J Obes.

[6] Frank LD, Andresen MA, Schmid TL (2004). Obesity relationships with community design, physical activity, and time spent in cars. Am J Prev Med.

[7] Sachs JD, Baillie JE, Sutherland WJ, Armsworth PR, Ash N, Beddington J (2009). Biodiversity conservation and the millennium development goals. Science.

[8] Snowdon W (2011). Challenges of noncommunicable diseases in the Pacific Islands: the need for evidence and data. Asia Pac J Public Health.

[9] World Health Organization (2010). World health statistics 2010.

[10] Swinburn B, Millar L, Utter J, Kremer P, Moodie M, Mavoa H (2011). The Pacific Obesity Prevention in Communities project: project overview and methods. Obes Rev.

[11] Swinburn BA, Sacks G, Hall KD, McPherson K, Finegood DT, Moodie ML (2011). The global obesity pandemic: shaped by global drivers and local environments. The Lancet.

[12] Tagoe HA, Dake FA (2011). Healthy lifestyle behaviour among Ghanaian adults in the phase of a health policy change. Glob Health.

[13] Singh KN, Sendall MC, Gurung A, Carne P. Understanding socio‐cultural influences on food intake in relation to overweight and obesity in a rural indigenous community of Fiji Islands. Health Promotion Journal of Australia. 2020. 10.1002/hpja.397 32761937

[14] World Health Organization. What is physical activity? Geneva: World Health Organization; 2018 [updated 23 February. Available from: https://www.who.int/news-room/fact-sheets/detail/physical-activity.

[15] Ministry of Health Fiji, World Health Organization. NCD Risk Factors STEPS REPORT 2011. Suva Fiji Islands: Fiji Goverment; 2015.

[16] Singh KN, Sendall M, Crane P, Fleming M. Understanding the socio-cultural context of obesity in rural iTaukei Fiji: ‘a participatory research approach’: Queensland University of Technology; 2017.

[17] Thompson SL, Chenhall RD, Brimblecombe JK (2013). Indigenous perspectives on active living in remote Australia: a qualitative exploration of the socio-cultural link between health, the environment and economics. BMC Public Health.

[18] Craike M, Wiesner G, Hilland TA, Bengoechea EG (2018). Interventions to improve physical activity among socioeconomically disadvantaged groups: an umbrella review. Int J Behav Nutr Phys Act.

[19] Hassen HY, Ndejjo R, Musinguzi G, Van Geertruyden J-P, Abrams S, Bastiaens H (2021). Effectiveness of community-based cardiovascular disease prevention interventions to improve physical activity: a systematic review and meta-regression. Prev Med.

[20] Pelletier CA, Smith-Forrester J, Klassen-Ross T (2017). A systematic review of physical activity interventions to improve physical fitness and health outcomes among Indigenous adults living in Canada. Preventive medicine reports.

[21] Lansbury Hall N, Crosby L (2022). Climate change impacts on health in remote indigenous communities in Australia. Int J Environ Health Res.

[22] Allender S, Nichols M, Foulkes C, Reynolds R, Waters E, King L (2011). The development of a network for community-based obesity prevention: the CO-OPS Collaboration. BMC Public Health.

[23] Rifkin SB. Community participation in maternal and child health/family planning programmes: an analysis based on case study materials. 1990.

[24] Finlay E, Kidd J. 16 Unpacking the ‘truth’ about the health gap: decolonising methodologies, cultural archives and the national aboriginal and torres Strait Islander health plan 2013–2023. BMJ Open. 2021;11(Suppl 1):A20-A.

[25] Harris R, Cormack D, Tobias M, Yeh L-C, Talamaivao N, Minster J (2012). Self-reported experience of racial discrimination and health care use in New Zealand: results from the 2006/07 New Zealand Health Survey. Am J Public Health.

[26] HealthInfoNet AI. Overview of Australian Indigenous health status 2014. 2015.

[27] Bell R, Tumilty S, Kira G, Smith C, Hale L (2016). Using a community based participatory research model within an indigenous framework to establish an exploratory platform of investigation into obesity. Obesity Medicine.

[28] Israel BA, Schulz AJ, Parker EA, Becker AB (2001). Community-based participatory research: policy recommendations for promoting a partnership approach in health research. Education for health.

[29] Minkler M, Wallerstein N. Community-based participatory research for health: From process to outcomes: Wiley. com; 2010.

[30] Agne AA, Daubert R, Munoz ML, Scarinci I, Cherrington AL. The cultural context of obesity: Exploring perceptions of obesity and weight loss among Latina immigrants. Journal of immigrant and minority health / Center for Minority Public Health. 2012;14(6). 10.1007/s10903-011-9557-3 PMC3818086 22130571

[31] Allen W, Kilvington M, Horn C. Using participatory and learning-based approaches for environmental management to help achieve constructive behaviour change. New Zealand: Landcare Research Contract Report, Ministry for the Environment W, New Zealand; 2002. Report No.: LC0102/057.

[32] Allender S, Cowburn G, Foster C (2006). Understanding participation in sport and physical activity among children and adults: a review of qualitative studies. Health Educ Res.

[33] Banks S, Manners P. Community-based participatory research: a guide to ethical principles and practice United Kingdom: Univeristy of Durham; 2011 [Available from: https://www.dur.ac.uk/resources/beacon/CBPREthicsGuidewebNovember20121.pdf.

[34] Jumper-Reeves L, Dustman PA, Harthun ML, Kulis S, Brown EF (2014). American Indian cultures: how CBPR illuminated intertribal cultural elements fundamental to an adaptation effort. Prev Sci.

[35] The Kellogg Health Scholars Program. Community-based participatory research (CBPR) and relationships between academe, community, policy, and public health practice.: Kellogg health Scholars; 2006 [updated 07 June 2016. Available from: http://www.kellogghealthscholars.org/about/community.php.

[36] Holt CM, Fawcett SB, Schultz JA, Jones JA, Berkowitz B, Wolff TJ (2013). Disseminating online tools for building capacity mong community practitioners. J Prev Interv Community.

[37] Windsor LC (2013). Using concept mapping in community-based participatory research: a mixed methods approach. J Mixed Methods Res.

[38] World Health Organization. Obesity and Overweight Fact Sheet Geneva, Switzerland: World Health Organization; 2016 [cited 2016 11 November]. Available from: http://www.who.int/mediacentre/factsheets/fs311/en/.

[39] Creswell J (2013). Qualitative, quantitative, and mixed methods approaches.

[40] Fiji Ministry Of Health And Medical Services. Health Information, Research and Analysis 2015 [Available from: https://www.health.gov.fj/health-information-research-and-analysis/.

[41] Smith J, Firth J (2011). Qualitative data analysis: the framework approach. Nurse Res.

[42] Swinburn B, Egger G, Raza F (1999). Dissecting obesogenic environments: the development and application of a framework for identifying and prioritizing environmental interventions for obesity. Prev Med.

[43] Lacey A, Luff D. Qualitative research analysis. United Kingdom: The NIHR RDS for the East Midlands/Yorkshire & the Humber; 2007.

[44] Dodgson JE (2019). Reflexivity in qualitative research. J Hum Lact.

[45] Hackett A, Strickland K. Using the framework approach to analyse qualitative data: a worked example. Nurse Researcher. 2018;26(3). 10.7748/nr.2018.e1580 30215482

[46] Anderson K, Cidro J. Decades of doing: indigenous women academics reflect on the practices of community-based health research. Journal of Empirical Research on Human Research Ethics. 2019:1556264619835707. 10.1177/1556264619835707 31018813

[47] Baba T, Boladuadu Ela, Vatuloka Wv, Nabobo-Baba U. Na Vuku Ni Vanua – Wisdom of the land: aspects of Fijian knowledge culture and history. Suva, Fiji: Native Academy Publishers, Institute of Indigenous Studies & Fijian Teachers Association Education; 2013.

[48] Belon AP, Nieuwendyk LM, Vallianatos H, Nykiforuk CIJ (2016). Community lenses revealing the role of sociocultural environment on physical activity. Am J Health Promot.

[49] Whitehead D (2003). Evaluating health promotion: a model for nursing practice. J Adv Nurs.

[50] Pyett P (2002). Working together to reduce health inequalities reflections on a collaborative participatory approach to health research. Aust N Z J Public Health.

[51] Ricciardelli LA, McCabe MP, Mavoa H, Fotu K, Goundar R, Schultz J (2007). The pursuit of muscularity among adolescent boys in Fiji and Tonga. Body Image.

[52] Becker AE, Gilman SE, Burwell RA (2005). Changes in prevalence of overweight and in body image among Fijian women between 1989 and 1998. Obes Res.

[53] Wate JT, Snowdon W, Millar L, Nichols M, Mavoa H, Goundar R (2013). Adolescent dietary patterns in Fiji and their relationships with standardized body mass index. Int J Behav Nutr Phys Act.

[54] Macdonald D, Abbott R, Jenkins D (2012). Physical activity of remote Indigenous Australian women: A postcolonial analysis of lifestyle. Leis Sci.

[55] Nash S, Arora A (2021). Interventions to improve health literacy among Aboriginal and Torres Strait Islander Peoples: a systematic review. BMC Public Health.

[56] Wicklum S, Willis E, Amson A, McGuire KA, Crowshoe LL, McBrien K (2021). A systematic literature review of physical activity-based health programs for Indigenous women: impacts on physical activity levels, obesity, and community building. SAGE Open.

[57] Thorp AA, Owen N, Neuhaus M, Dunstan DW (2011). Sedentary behaviors and subsequent health outcomes in adults: a systematic review of longitudinal studies, 1996–2011. Am J Prev Med.

[58] Israel B, Coombe CM, Cheezum RR, Schulz AJ, McGranaghan RJ, Lichtenstein R (2010). Community-based participatory research: a capacity-building approach for policy advocacy aimed at eliminating health disparities. Am J Public Health.

[59] Wallerstein, Oetzel J, Duran B, Tafoya G, Belone L, Rae R. What predicts outcomes in CBPR. Community-based participatory research for health: from processes to outcomes. 2 ed. San Francisco, USA: Jossey-Bass; 2008. 371–88 p.

[60] Walters KL, Stately A, Evans-Campbell T, Simoni JM, Duran B, Schultz K, Stiffman A (2009). 'Indigenist' collaborative research efforts in Native American communities. The field research survival guide.

[61] Minkler M (2010). Linking science and policy through community-based participatory research to study and address health disparities. Am J Public Health.

[62] Government NT. Health promotion strategic frramework 2011 - 2015. In: Health Mo, editor. http://www.health.nt.gov.au/library/scripts/objectifyMedia.aspx?file=pdf/66/68.pdf: Northern Territory Government; 2015.

[63] Alzahrani H, Mackey M, Stamatakis E, Pinheiro MB, Wicks M, Shirley D (2019). The effectiveness of incidental physical activity interventions compared to other interventions in the management of people with low back pain: a systematic review and meta-analysis of randomised controlled trials. Phys Ther Sport.

